# Variation in Volatile Flavor Compounds of Cooked Mutton Meatballs during Storage

**DOI:** 10.3390/foods10102430

**Published:** 2021-10-13

**Authors:** Yu Zhang, Yuwei Sun, Huanlu Song

**Affiliations:** Laboratory of Molecular Sensory Science, College of Food and Health, Beijing Technology and Business University (BTBU), Beijing 100048, China; zhang_yu@btbu.edu.cn (Y.Z.); m15827192560@163.com (Y.S.)

**Keywords:** SPME, SAFE, cooked mutton meatballs, storage, key odorant active compounds, sensory evaluation, PLSR

## Abstract

Solid phase microextraction (SPME) and Solvent-Assisted Flavor Evaporation (SAFE) were used to analyze the flavor changes of cooked mutton meatballs during storage by gas chromatography-olfactometrymass spectrometry (GC-O-MS), sensory evaluation and Partial Least Squares Regression (PLSR). With the increase of storage time, the concentrations of various volatile compounds in cooked mutton meatballs decreased to varying degrees at the later stage of storage, indicating that the aroma was gradually weakened, which was consistent with the results of sensory evaluation. At 30 days of storage, the overall aroma profile was more prominent, and at the later stage of storage, the sulfur odor was more prominent. The correlation of PLSR further confirmed the credibility of the results. Compared with the SPME and SAFE extraction methods, SPME extracted more flavor substances, and the SAFE extraction rate was higher, which indicated that the combination of several methods was needed for aroma extraction. An analysis of the dilution results and odor activity value (OAV) showed that the key aroma components during storage were 1-octene-3-ol, linalool, methylallyl sulfide, diallyl disulfide, 2-pinene, hexanal and butyric acid.

## 1. Introduction

Mutton is a traditional medicine and food supplement in China that has rich nutritional value, high protein content and low cholesterol [1]. Tan mutton is a characteristic and advantageous animal breed in Ningxia Province [2]. The meat balls made by Tan sheep are fresh and tender, low in fat and are not goat-like [1,3]. As a traditional meat product in China, meatballs are popular among consumers because of their availability and unique taste [1]. Storage has a great impact on the quality of meat products [4]. Chunha et al. [5] reviewed the mechanisms associated with lipid and protein oxidation and their implications on meat quality attributes, as oxidative damage is one of the main reasons for loss of quality in sheep and goat meat and meat products in general. Xiao et al. [6] also reviewed that oxidation in lipid and protein fractions of meat has been demonstrated as the main, non-microbial cause of quality deterioration during processing. This is because lipids and proteins in meat are easily susceptible to oxidative damages due to the rapid depletion of endogenous antioxidants after slaughter. Pinheiro et al. [7] reported that the lipid oxidation increased over time during frozen storage. After storage for a period of time, the oxidation of fat, degradation of amino acids and occurrence of Maillard reaction (reaction between amino acid and reducing sugar, that is, carbonyl-ammonia reaction) will make meat taste better [8,9]. However, some studies have found that odorous oxides, such as aldehydes and ketones, will be generated in the adipose tissue of meat products after excessive oxidation [10]. Excessive storage time is a factor of excessive oxidation, and the product will have unpleasant odor, which will affect its quality [3]. Because the storage of meat products can cause various problems, research on storage periods has emerged in an endless stream in recent years. Cooked mutton balls have a high moisture content, and problems such as fat oxidation, decomposition and deterioration are easy to happen during storage, which will lead to the loss of flavor and will reduce the costumer’s desire to purchase the products [11,12]. Understanding the variations in compounds responsible for the flavor of meatballs during storage can provide the theoretical basis for improving product quality by controlling the processes that control the formation of these compounds. Previously, we reported the variation of aroma components during frozen storage of cooked beef balls by SPME/SAFE/GC-O-MS; some results are interesting [13].

At present, there are few studies on meatballs, most of which focus on the improvement of meatball quality and the optimization of processing technology. Parvin et al. [14] studied the effects of four different heating methods on the pH, color, texture, antioxidant content and overheated flavor of beef meatballs and studied the lipid and protein oxidation levels of four kinds of beef meatballs with different extracts, so as to improve the overall acceptability of the meatballs. Parvin et al.’s study was to evaluate the effects of different levels of olive leaf extract on fresh and pickled mutton meatballs. Parvin et al. [15] showed that 0.3% olive leaf extract could be used as a natural antioxidant and had a positive effect on the quality of lamb meatballs. Özturk et al. [16] studied the physicochemical properties of pumpkin kernel powder and determined that adding 3% pumpkin kernel powder can improve the distribution of fatty acids and reduce the fat content of beef meatballs. Zhang et al. [17] used headspace solid-phase microextraction (HS-SPME) and gas chromatography-mass spectrometry (GC-MS) to identify and analyze the volatile compounds in three different cooking methods of Mashanshan mutton in northern Guizhou. The results showed that hexanal, nonanal, octanol, limonene and caryophyllene were the main components of cooked mutton flavor. Zhan et al. [3] used volatile compound fingerprinting technology and stoichiometry to control the quality of mutton, and 4-ethyleneic acid, 4-methylnonanoic acid and caproic acid were identified as the decisive characteristic flavor compounds of mutton.

In the present study, solid-phase microextraction (SPME) and solvent-assisted flavor evaporation (SAFE) extraction methods were used to study the changes of volatile flavor compounds in cooked mutton meatballs during storage by gas chromatography-olfactometry-mass spectrometry (GC-O-MS). Similarities and differences between the SPME and SAFE extraction methods were compared to find out the variation regularity of volatile compounds in cooked mutton meatballs during storage. The aroma dilution analysis combined with odor activity value (OAV) was used to determine the key odor-active substances of cooked mutton meatballs during storage. Finally, PLSR (Partial Least Squares Regression) was used to explain the relationship between odor-active compounds, sensory evaluation and storage period.

## 2. Materials and Methods

### 2.1. Chemicals

Chromatography standards, including 2-methyl-3-heptanone (purity > 99%), n-alkanes (C_7_–C_30_) (purity > 99%), n-hexane (purity > 99%) and n-pentane (purity > 99%), were purchased from Sigma-Aldrich (St. Louis, MO, USA). Analytical reagents, including diethyl ether (purity > 99%), n-pentane (purity > 99%) and anhydrous sodium sulfate (purity > 99%) were provided by Yifengtiancheng Scientific Instruments Co. Ltd. (Beijing, China). Nitrogen (purity > 99.99%) was supplied by Beijing Haipubeifen Gas Industry Co. Ltd. (Beijing, China)

### 2.2. Cooked Mutton Meatballs Samples

Tan mutton (500 g) from Ningxia was minced by a meat grinder, and the remaining ingredients were added according to the list of ingredients (water 20.00%, salt 2.40%, garlic 1.00%, sugar 2.00%, MSG (monosodium glutamate) 3.00%, potassium tripolyphosphate 0.40%, black pepper 0.01%, muscade 0.02%, ginger powder 0.02%). Once the minced meat was mixed, it was then put in a refrigerator at 4 °C for half an hour. At the end of the time, the minced meat was taken out of the refrigerator and then made into meatballs by hand with a diameter of about 20 mm. After the water was boiled to 60 °C, the meatballs were put into the pot and cooked at 90 °C for 10 min. After cooking, the mutton meatballs were quickly put into a freezer at −20 °C for a storage of 0 days, 30 days, 60 days, 90 days.

### 2.3. Sensory Evaluation

The tested sample (15 g) was cut into small pieces and placed in a 40 mL headspace vial. The aroma profile was evaluated by 12 trained panelists who were recruited from the Laboratory of Molecular Sensory Science, Beijing Technology and Business University (Beijing, China). They all went through a week of training before evaluation. Flavor attributes were described following a discussion among the panelists, including spicy, milky, meaty, sulfur and goat-like. The specific description and training criteria for each attribute are as follows: garlic, similar to the aroma of garlic; spicy, similar to the aroma of a blend of spices; meaty, similar to the aroma of cooked mutton meatballs; sulfur, similar to the odor of diluted 2-methylthiophene; goat-like, the special odor of mutton. The assessment is based on a scale of 11, from 0 to 10.

### 2.4. Solid-Phase Microextraction (SPME)

The frozen sample of cooked mutton meatballs was thawed in the refrigerator overnight, and a blender was used to break it up. The cooked mutton meatballs (5 g) were weighed accurately, put into a 40 mL headspace vial (Gerstel, Germany) and 1 μL 2-methyl-3-heptanone (0.816 g/L in hexane) was added as an internal standard. Then, the vial was put into constant temperature water bath (HH-1, Guohua Company, China) at 60 °C for 20 min. A solid-phase microextraction sampler with a divinylbenzene/carboxen/polydimethylsiloxane (DVB/CAR/PDMS) fiber (50/30 μm, Supelco, Bellefonte, PA, U.S.A.) was used to extract the volatile compounds in headspace for 40 min at 60 °C. The extracted compound was thermally desorbed in the injection port of the GC-O-MS instrument at 250 °C for 5 min for gas chromatography-olfactometry-mass spectrometry (GC-O-MS) (7890A-7000B equipped with EI ion source and NIST 08 database, Agilent Technologies, CA, USA; Sniffer 9000, Brechbuhler, Switzerland) analysis. [13,18]

### 2.5. Solvent-Assisted Flavor Evaporation (SAFE)

The frozen sample of cooked mutton meatballs was also treated as described in Section 2.4. The 30 g sample of beef ball was accurately weighed and added with 80 mL anhydrous ethyl ether and 40 mL n-pentane. An aliquot of 1 μL 2-methyl-3-heptanone (816 μg/μL) was added as an internal standard. Solvent extraction was performed using the SAFE apparatus (Deutsche Forschungsanstalt für Lebensmittelchemie, Freising, Germany), as described by Engel [19]. A high vacuum (10^−4^ to 10^−5^ Pa) was achieved by a combination system of a vacuum pump and a turbine pump (Edwards, UK). The water bath was maintained at 40 °C, and liquid nitrogen was utilized to maintain a very low temperature (−196 °C) for the trapping of the extracted and separated volatile substances. The extract was dried by anhydrous sodium sulfate (Na_2_SO_4_) and concentrated to approximately 10 mL by a Vigreux column and finally to about 500 μL under a gentle flow of nitrogen (99.9992% purity). All analyses were repeated in triplicate.

### 2.6. Gas Chromatography−Olfactometry−Mass Spectrometry Analysis

A gas chromatography−mass spectrometry (GC-MS) instrument (7890A-7000B, Agilent Technologies, Inc., Santa Clara, CA, USA) equipped with an olfactory detection port (Sniffer 9000, Brechbuhler, Schlieren, Switzerland) was used to analyze and identify the aroma compounds. The separation of volatile mixture was carried out through a gas chromatography (GC) capillary column (30 m × 0.25 mm, 0.25 μm, J & W Scientific, Folsom, CA, USA); the temperature of the GC inlet was 250 °C. The initial column temperature was 40 °C, maintained for 4 min, increased to 70 °C at 6 °C/min, then increased to 240 °C at 8 °C/min and maintained for 5 min. The carrier gas is helium, the column flow rate is 0.8 mL/min and the mode of injection inlet is splitless. The interface temperature of the mass spectrometer (MS) was 250 °C, and electron collision is produced at an ionization energy of 70 ev in a mass to charge ratio of 50~350 m/z. The mass spectrum source temperature is 230 °C.

### 2.7. Qualitative Analysis

All volatile compounds were identified after comprehensive evaluation by comparing the results with the NIST 08 spectrum library, standard compound retention index (*RI*) and olfactory-sniffing (O). *RI* value is calculated according to the peak retention time of the target and the peak retention time of the series of alkanes under the same temperament condition [20]. The calculation formula is as follows:

(1) $$RI=100N+100n\frac{t_{R_{a}}-t_{R_{N}}}{t_{R_{\left(N+n\right)}}-t_{R_{N}}}$$

*N*: the number of carbon atoms in a smaller normal alkane; *n*: the number of carbon atoms in the n-alkane with the retention time larger than the retention time of the target compound; $t_{R_{a}}$: retention time of unknown, $t_{R_{N}}$: retention time of n-alkanes with a small number of carbon atoms, $t_{R_{\left(N+n\right)}}$: retention time of a large number of carbon atoms.

### 2.8. Quantitative Analysis

For the purpose of the semi-quantification of target compounds, 2-methyl-3-heptanone (soluble in n-hexane) with a concentration of 0.816 g/L was used as the internal standard. The ratio of the peak area of the unknown odor substance and the internal standard substance in the sample is multiplied by the concentration of the internal standard substance to obtain the concentration of the unknown odor substance

### 2.9. Aroma Extraction Dilution Analysis

AEDA method is used to dilute volatile components gradually by solvent until the aroma of flavor compounds cannot be detected at the sniffing port. Key odor-active compounds can be screened out by this method. Two types of dilution analysis were performed in this study. The first one is AEDA (aroma extract dilution analysis), a solvent extraction method similar to that described by Wang. [21] The ratio of the serial solvent dilutions performed was 1:3. The importance of each volatile component is determined by the flavor dilution factor (FD factor), which is the highest number of odor-active substances detected. The flavor dilution (FD) factor was the highest dilution multiple of the compound, and the FD factors were expressed as FD (1, 3, 9 and so on), representing the serial dilutions (1:3, 1:9, 1:27 and so on, respectively). The second one is DHDA, that is, dynamic headspace dilution analysis, similar to that described by Kim and Ferreira. [22,23] The dilution ratio was achieved by changing the split ratio of GC-O by 1:1, 3:1, 9:1, 27:1, 81:1 and so on. The corresponding FD factors were 0, 1, 2, 3 and 4. The FD values were obtained from five experienced sensory panelists who sniffed the aroma compounds as separated by GC-MS (described below). All FD values were averaged and rounded to the next whole number.

### 2.10. Odor Activity Value

This method was used to further indicate which of the compounds revealed by AEDA actually contribute to the aroma of cooked mutton meatballs. OAV is the ratio of the content of volatile compounds (μg/kg) to the threshold value of volatile compounds in water (μg/kg) [24,25].

### 2.11. Statistical Analysis

The radar chart and bar chart are compiled in Microsoft Excel 2016. IBM SPSS Statistics 22 software was used for the data analysis of single factor variance (ANOVA) and Duncan’s Multi-Range test. Each experiment was repeated three times under the same conditions, and the standard deviation was calculated for the results of the three times. At *p* < 0.05, the data were considered statistically significant. The flavor substances and sensory properties of cooked mutton meatballs with different storage times were analyzed with Unscrambler Version 9.7 statistical analysis software, and the correlation between flavor substances and sensory properties was analyzed by partial least squares regression (PLSR).

## 3. Results

### 3.1. Comparison of SPME and SAFE Extraction Methods

The Solid-Phase Microextraction (SPME) method combined with Gas Chromatography-Olfactometry-Mass Spectrometry (GC-O-MS) was used to extract and detect volatile flavor substances from cooked mutton meatballs (freshly cooked, not frozen). Eighty-nine different kinds of volatile compounds were identified, as shown in Table 1, including 12 alcohols, 6 sulfur-containing compounds, 25 olefins, 15 aromatic compounds, 7 aldehydes, 6 esters, 3 ketones and 15 terpenes. During storage, there were 43 kinds of volatile compounds, including 7 alcohols, 3 sulfur-containing compounds, 11 olefin compounds, 5 aromatic compounds, 3 aldehydes, 4 esters, 1 ketone and 9 terpenoids. Among all the volatile compounds detected, 36 odor compounds could be smelled, including 4 alcohols, 5 sulfur-containing compounds, 11 olefin compounds, 6 aromatic compounds, 3 aldehydes, 1 ketone and 7 terpenes.

Similar to Solid-Phase Microextraction (SPME), the SAFE method was also applied combined with Gas Chromatography−Olfactometry−Mass Spectrometry (GC-O-MS) for the extraction and identification of the volatile flavor substances of cooked mutton balls, and 58 kinds of odor-active compounds were identified. The results are shown in Table 2, including 9 alcohols, 3 sulfur compounds, 6 olefins, 10 aromatic compounds, 3 esters, 5 aldehydes, 1 ketone, 12 terpenoids and 5 phenols. During storage, there were 27 different kinds of volatile compounds identified, including 6 alcohols, 2 sulfur-containing compounds, 2 olefin compounds, 5 aromatic compounds, 2 aldehydes, 2 esters, 1 ketone, 3 terpenes and 4 phenols. Among them, 23 odor compounds could be smelled, including 5 alcohols, 2 sulfur-containing compounds, 2 olefin compounds, 3 aromatic compounds, 2 aldehydes, 1 ketone, 7 terpenes and 1 phenol.

**Figure 1:**
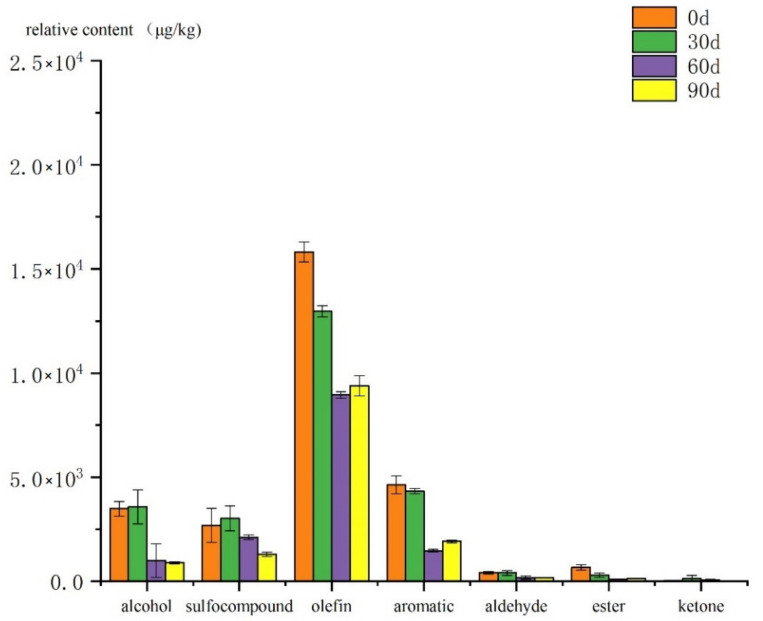
Changes in various aroma compounds detected by SPMEGC-O-MS during storage.

**Figure 2:**
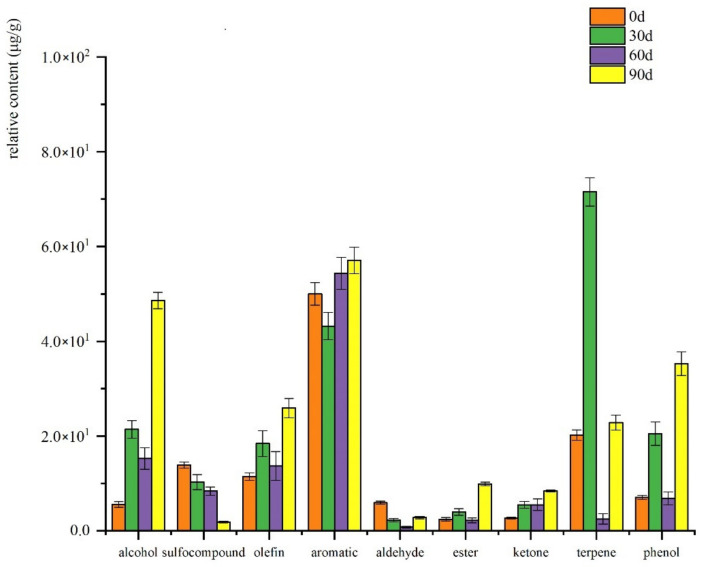
Changes in various aroma compounds detected by SAFE GC-O-MS during storage.

The changes of various volatile compounds in cooked mutton meatballs during storage were analyzed by SPME/GC-O-MS, as shown in Figure 1. At 0 days, the relative contents of alcohols, sulfur-containing compounds, olefin, aromatic compounds, aldehydes, esters, ketones and terpenes are the highest, and at the later stage of storage, the concentrations of all volatile compounds decreased significantly. SAFE/GC-O-MS was used to analyze the changes of various volatile compounds during the storage period of cooked mutton meatballs. As shown in Figure 2, most volatile compounds had a higher concentration in the late storage period, and the overall change of aromatic compounds was little during the storage period.

The SPME and SAFE extraction methods were compared, and 89 volatile compounds were identified by SPME and 58 by SAFE. A total of 34 volatile compounds were identified by SPME and SAFE together, including 1-octene-3-ol, linalool, terpinen-4-ol, diallyl sulfide, allyl methyl disulphide, α-pinene, 3-carene, anethole, eugenol, n-hexanal and benzaldehyde. The SPME method extracted more volatile compounds than SAFE, and more odorous compounds were smelled. In the SPME method, odor-active compounds accounted for 36.36%, 35.06%, 41.8% and 36.68% (0d, 30d, 60d, 90d) of the total volatile compounds, while in SAFE, odor-active compounds accounted for 36.36%, 35.06%, 41.8% and 36.68% of the total volatile compounds. During the whole storage period, the proportion of odor-active compounds extracted by SAFE was slightly higher than that by SPME, indicating that solvent extraction had a higher extraction rate than headspace extraction, which was consistent with our previous results [13]. The number and concentration of volatile compounds extracted by SPME and SAFE were different; SAFE has a good extraction effect on phenolic compounds and can extract more heterocyclic compounds. To sum up, the SPME extraction fiber layer has different adsorption capacity to substances, and SAFE will have solvent loss in the extraction process, so the volatile compounds extracted by the two methods are not consistent. SPME and SAFE are different: SPME is a headspace analysis method, while SAFE is solvent extraction. [26] From the overall effect of this study, SPME is better than SAFE. By combining the two extraction methods, the results will be more comprehensive and complete. 

### 3.2. Analysis of Volatile Flavor Compounds in Cooked Mutton Meatballs during Storage

It can be seen from Figure 1 and Figure 2 that the content of hydrocarbon substances is high in various volatile compounds during each storage period. Olefins generally have a higher odor threshold and do not contribute much to the overall aroma. However, like aldehydes, they are both products of fatty acid breakdown and intermediates of heterocyclic substances, so the variation trend of their concentration has a certain correlation with the presentation of flavor [27]. Olefins are mostly related to spices, such as α-copaene and 3-carene, which are among the main components of turpentine and have the aroma of turpentine. 1-methyl-4-(6-methylhept-5-en-2-yl)benzene is found in codonopsis, dried ginger and American ginseng. Sulphur-containing compounds such as allyl methyl sulfide, diallyl sulfide, allyl methyl disulfide, diallyl disulfide and garlic are related components. Garlic is used to make the mutton meatballs, giving them a strong garlic smell and helping to mask the goat-like odor. Whether SPME or SAFE, sulfur compounds have a higher proportion and concentration in the total volatiles. By SPME/GC-O-MS, seven kinds of volatile sulphur-containing compounds were extracted; of them, four kinds could be smelled. By SAFE/GC-O-MS, four kinds were extracted and three kinds were smelled, indicating that sulfur compounds contribute more to the overall aroma components of cooked mutton meatballs. Aldehydes are usually the products of oxidative degradation of fat, and the volatile compounds of aldehydes in cooked mutton meatballs are not high. Although the relative content is not high, the threshold value of aldehydes is low, which contributes to the overall degree of mutton meatball [28]. Hexanal and nonanal can be smelled by SPME and SAFE/GC-O-MS, which is consistent with the research results of the characteristic aroma components of Ningxia Tan mutton [29]. Both SPME and SAFE/GC-O-MS analysis showed a decrease in the relative content of aldehydes at the later stage of storage, indicating that flavor loss was relatively serious at the later stage of storage, which was consistent with the results of sensory evaluation. Alcohol compounds may be produced by the microbial metabolism of glucose and amino acids or may be caused by the oxidation of fatty acids. The threshold value of alcohol compounds is generally high, and it does not contribute much to the flavor of cooked lamb meatballs during storage [29]. Esters and ketones were relatively high in cooked mutton meatballs, but the volatile compounds retrieved were not smelled, which contributed little to the overall aroma of cooked mutton meatballs [30]. Phenols were detected only in SAFE/GC-O-MS, and their proportion gradually increased from 5.68% to 14.84% in the storage process. Among the five detected phenolic substances, only 2,6-ditert-butyl-4-methylphenol could be smelled, but the intensity of the smell was not large. The key odor-active substances in cooking mutton meatballs during different storage periods were identified.

Among the many volatile compounds in cooked mutton meatballs, only a small part of them play an important role in contributing to the overall flavor, and these odor-active compounds are the main aroma components in cooked mutton meatballs. In order to further determine the degree of aroma contribution to the storage period, the flavor dilution factor of the odor-active compound, namely the FD factor, can be obtained by gradual dilution. The larger the FD factor, the greater the contribution of the odor-active compound to the cooked mutton meatballs is. The FD factor is used to evaluate how much these flavor compounds contribute to the overall aroma.

It can be seen from Table 3 and Table 4 that, among the volatile flavor components extracted by SPME, the number of compounds that could be smelled were 21, 21, 17 and 13. While for SAFE, the number of odor-active compounds identified were 13, 24, 20 and 12. The number of volatile compounds was much higher than that of odor-active compounds, indicating that most of the volatile compounds had no significant effect on the formation of the aroma of cooked mutton meatballs.

During the storage of cooked mutton meatballs, the increase and decrease of odor-active compounds and the FD factor are also constantly changing. Compared with the change of FD factor in cooked mutton meatballs during storage, it can be seen that the number of odor-active compounds identified by SPME/GC-O-MS is gradually decreasing. The FD factors of allyl mercaptan (garlic), 1-octene-3-alcohol (mushroom), linalool (floral), diallyl disulfide ether (onion), diallyl trisulfide (onion), α-pinene (pine), 3-carene (resin), d-cadinene (medicine), unknown (sweet) and ethyl caprate (grape) were higher during the storage period, which contributed to the overall aroma degree of cooked mutton meatballs. However, the FD factors of caryophylene (spice), hexanal (grassy), allyl methyl disulfide (onion), diallyl disulfide (onion), linalool (floral), geraniol (fruity), unknown-(balsam) and butyric acid (sweaty) during the storage were higher SAFE during the storage period, which contributed to the overall aroma degree of cooked mutton meatballs.

Table 5 lists odor-active compounds with OAV values greater than 1 by SAFE and SPME/GC-O-MS. OAV is not only related to the concentration of volatile flavor substances but also to its odor threshold. The greater the OAV value is, the greater the contribution to the whole sample is. It is worth noting that the FD factor of odor-active substances with an OAV value greater than 1 was too low for β-pinene, nonanal, ((1S)-endo)-(-)-borneol and 2-methylnaphthalene. The FD factors of 3-carene, d-cadinenee, isosafcamphor, ethyl caprate and unknown were high, but the OAV values were less than 1 or no, indicating that the two methods still had differences. By comparing OAV with the dilution analysis results, it can be concluded that the OAV of 1-octene-3-ol, linalool, methylallyl sulfide, diallyl disulfide, 2-pinene, hexanal and butyric acid is greater than 1 and that the FD factor is large, which are the key odor-active components of cooked mutton meatball during storage.

### 3.3. Sensory Evaluation

**Figure 3:**
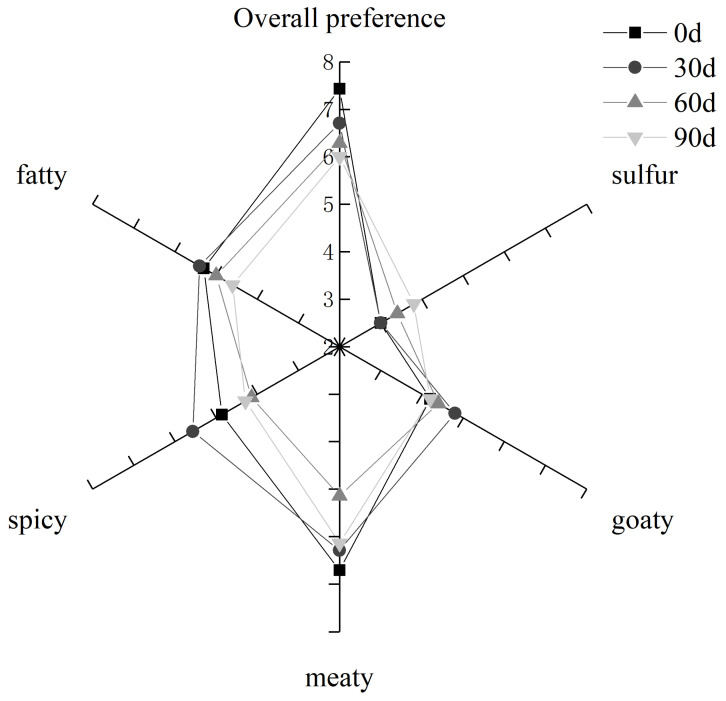
Sensory evaluation of cooked mutton meatballs.

The sensory evaluation results of cooked mutton meatballs are shown in Figure 3. It can be seen from the figure that the overall preference gradually decreases with the extension of the storage period

However, when stored for 30 days, the odor of the meat, the spices and its goat-like nature becomes more prominent. After that, the overall aroma profile becomes weak as the storage period prolongs—that is, the aroma of cooked mutton meatballs begins to weaken.

### 3.4. PLSR (Partial Least Squares Regression) Analysis of Different Storage Times

**Figure 4:**
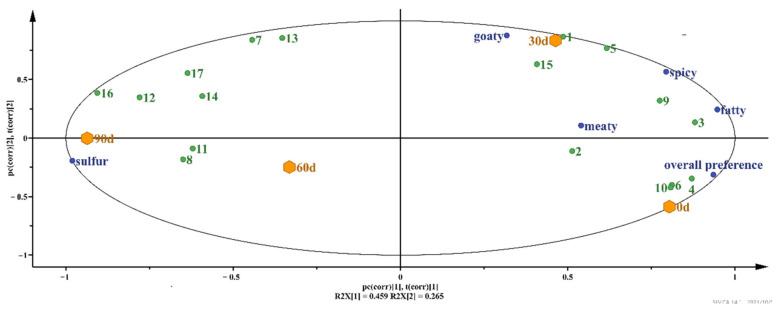
The correlations among the sensory evaluation and storage time in cooked mutton meatball by PLSR analyzing.

In order to comprehensively analyze the correlations among the key odorant active compounds, sensory evaluation and storage time, PLSR (Partial Least Squares Regression) method was employed to analyze the correlations. The X axis of odor-active compounds, the Y axis of sensory evaluation and the different storage times were used for analysis. As shown in Figure 4, for the first principal component, unpleasant odors such as sulfur and goat-like, which are stored for 60 days and 90 days, are concentrated in the positively correlated region of the coordinate axis, while 0 days and 30 days storage, as well as the degree of preference, meat, spice and fat aroma, are located in the negatively correlated region of the coordinate axis. The results showed that there were significant differences between the early and late storage samples. Sensory properties and most volatile compounds are located between r^2^ = 0.5 and r^2^ = 1, suggesting that the PLSR can adequately account for the above variables. Samples located in the same quadrant were strongly correlated with sensory attributes. Cooked mutton meatballs stored for 30 days were strongly correlated with spice and fat, which was similar to the sensory analysis results in Figure 4. At 60 and 90 days of storage, there was a strong correlation with sulfur odor, indicating that odor was prominent at the later stage of storage, which was consistent with the results of sensory evaluation.

The green numbers is corresponding with the 17 key odor-active compounds in Table 5.

## 4. Discussion 

Solid phase microextraction (SPME) and solvent-assisted flavor evaporation (SAFE) combined with gas chromatography-olfactometry-mass spectrometry (GC-O-MS) were employed to analyze the flavor changes of cooked mutton meatballs during storage. A total of 89 volatile compounds were identified by SPME/GC-O-MS and 58 by SAFE/GC-O-MS. A total of 34 volatile compounds were identified by both SPME and SAFE/GC-O-MS. The quantity and concentration of volatile compounds decreased to different degrees at the later stage of storage. According to FD factors and OAVs, the key odor-active compounds of cooked mutton meatball during storage were 1-octene-3-ol (mushroom), linalool (flower), methyl allyl sulfide (onion), diallyl disulfide ether (onion), 2-pinene (pine oil taste), hexanal (grassy) and butyric acid (sweaty). PLSR analysis confirmed the correlation between sensory properties and flavor compounds. At 30 days of storage, the overall aroma profile was more prominent, and at the later stage of storage, the sulfur odor was more prominent. The correlation of PLSR further confirmed the credibility of sensory analysis. As this study reported the variation of the odor-active compounds of cooked mutton meatball during storage, it may provide some useful information for the improvement of the storage conditions for better meat product quality.

## Figures and Tables

**Table 1:** Volatile compounds identified by SPME GC-O-MS during the storage period of cooked mutton balls.

_N_o	RI	CAS No	Compounds	Perception	Identification Method	Relative Content (μg/kg)			
						0 d	30 d	60 d	90 d
alcohol									
1	1392	3391-86-4	1-Octen-3-ol	mushroom	RI/O/MS	51.97 ± 12.88 ^a^	52.0 ± 17.94 ^a^	59.55 ± 52.29 ^ab^	37.19 ± 0.92 ^b^
2	1495	78-70-6	Linalool	floral	RI/O/MS	338.78 ± 17.45 ^a^	304.01 ± 58.97 ^b^	118.93 ± 59.61 ^c^	163.67 ± 11.19 ^d^
3	1542	619-62-5	1-methyl-4-(propan-2-yl)cyclohex-2-en-1-ol	herbal	RI/MS	51.90 ± 12.90 ^a^	40.1 ± 4.10 ^b^	8.67 ± 0.61 ^c^	ND ^d^
4	1544	15537-55-0	unknown	balsam	RI/MS	333.72 ± 72.77 ^a^	262.57 ± 24.10 ^a^	57.87 ± 47.30 ^b^	34.88 ± 11.70 ^c^
5	1592	562-74-3	Terpinen-4-ol	pepper woody earth musty sweet	RI/MS	2538.45 ± 201.46 ^a^	2310.3 ± 523.04 ^b^	676.25 ± 590.84 ^c^	1197.41 ± 12.38 ^d^
6	1612	586-81-2	p-menth-4(8)-en-1-ol	terpineol lilac	RI/O/MS	35.26 ± 3.22 ^a^	39.4 ± 15.32 ^a^	52.14 ± 52.21 ^b^	33.25 ± 2.19 ^a^
7	1690	98-55-5	p-menth-1-en-8-ol	pine, citrus, woody, floral	RI/MS	ND ^a^	441.54 ± 147.51 ^b^	ND ^a^	218.86 ± 3.66 ^c^
8	1695	507-70-0	((1S)-endo)-(-)-borneol	pine, woody, camphor, balsamic	RI/MS	ND ^a^	ND ^a^	ND ^a^	43.72 ± 1.50 ^b^
9	1698	464-45-9	L(-)-Borneol	pine, woody, camphor	RI/MS	72.59 ± 8.79 ^a^	68.6 ± 15.78 ^b^	ND ^a^	ND ^a^
10	1753	106-22-9	(-)-β-citronellol	floral, leather, waxy, rose, bud citrus	RI/MS	26.83 ± 6.89 ^a^	27.9 ± 6.09 ^a^	6.05 ± 3.10 ^b^	11.22 ± 4.54 ^b^
11	1834	106-24-1	Geraniol	waxy	RI/O/MS	24.25 ± 7.75 ^a^	31.9 ± 9.48 ^b^	4.07 ± 1.93 ^c^	ND ^c^
12	2073	639-99-6	(1S,2S,4R)-(-)-α,α-dimethyl-1-vinyl-o-menth-8-ene-4-methanol	green, woody, spi-cy, rose	RI/MS	10.08 ± 4.60 ^a^	ND ^b^	ND ^b^	2.49 ± 0.22 ^a^
Sulfur compound									
13	871	870-23-5	Allyl mercaptan	garlic, onion	RI/O/MS	1307.61 ± 116.28 ^a^	1048.8 ± 109.27 ^a^	683.03 ± 171.19 ^b^	435.02 ± 37.73 ^c^
14	944	10152-76-8	Allyl methyl sulfide	garlic, onion	RI/O/MS	122.05 ± 11.25 ^a^	67.4 ± 8.54 ^b^	38.57 ± 11.95 ^c^	38.12 ± 6.46 ^c^
15	1126	592-88-1	diallyl sulfide	sulfurous onion garlic horseradish metallic	RI/MS	103.42 ± 42.97 ^a^	ND ^b^	ND ^b^	ND ^b^
16	1262	2179-58-0	allyl methyl disulphide	garlic, onion	RI/O/MS	ND ^a^	75.9 ± 65.85 ^b^	315.58 ± 238.35 ^c^	ND ^a^
17	1454	2179-57-9	Diallyl disulfide	garlic, onion	RI/O/MS	1115.71 ± 642.81 ^a^	1794.1 ± 397.58 ^b^	1069.36 ± 749.64 ^a^	824.12 ± 56.83 ^c^
18	1754	2050-87-5	Diallyl trisulfide	garlic green onion metallic	RI/O/MS	30.55 ± 8.51 ^a^	37.0 ± 10.14 ^a^	13.27 ± 3.79 ^b^	ND ^c^
olefin									
19	1013	80-56-8	α-Pinene	pine	RI/O/MS	1660 ± 46.03 ^a^	1040.2 ± 269.08 ^b^	764.83 ± 153.34 ^b^	842.45 ± 73.18 ^c^
20	1019	2867/5/2	3-Thujene	woody, green, herb	RI/MS	2623.09 ± 449.39 ^a^	2321.4 ± 607.24 ^a^	1873.77 ± 263.03 ^b^	1103.67 ± 110.19 ^c^
21	1047	471-84-1	unknown	camphor	RI/MS	9 ± 0.42 ^a^	7.0 ± 3.53 ^b^	7.48 ± 1.42 ^b^	4.69 ± 0.40 ^a^
22	1101	3387-41-5	Sabinene	woody, terpene, citrus, pine, spice	RI/MS	4032.92 ± 3660.72 ^a^	3705.8 ± 232.28 ^b^	2189.24 ± 537.18 ^c^	3273.35 ± 39.60 ^b^
23	1141	123-35-3	Myrcene	peppery terpene spicy balsam plastic	RI/MS	2397.03 ± 134.08 ^a^	1704.6 ± 510.91 ^b^	1382.91 ± 214.23 ^c^	1485.10 ± 95.96 ^c^
24	1158	99-86-5	p-mentha-1,3-diene	lemon	RI/O/MS	1418.14 ± 256.37 ^a^	1131.9 ± 371.75 ^b^	1111.79 ± 152.95 ^b^	760.87 ± 40.85 ^c^
25	1188	555-10-2	p-mentha-1(7),2-diene	terpentine	RI/O/MS	2087.99 ± 118.32 ^a^	1667.5 ± 297.74 ^b^	1309.34 ± 185.19 ^c^	1293.51 ± 36.65 ^c^
26	1214	3779-61-1	trans-Ocimene	sweet, herbal	RI/O/MS	14.61 ± 4.02 ^a^	10.6 ± 7.94 ^a^	8.05 ± 2.22 ^b^	ND ^c^
27	1233	3338-55-4	(Z)-β-Ocimene	warm floral herb flower sweet	RI/MS	64.4 ± 2.06 ^a^	ND ^b^	ND ^b^	ND ^b^
28	1244	100-42-5	Styrene	sweet balsam floral plastic	RI/MS	26.35 ± 10.64 ^a^	24.3 ± 6.64 ^a^	32.18 ± 8.15 ^b^	30.76 ± 9.50 ^b^
29	1418	18368-95-1	p-menthatriene, p-mentha-1,3,8-triene	turpentine	RI/O/MS	27.79 ± 8.32 ^a^	ND ^b^	ND ^b^	ND ^b^
30	1428	1195-32-0	α,para-Dimethylstyrene	clove	RI/O/MS	143.6 ± 21.5 ^a^	116.79 ± 32.31 ^b^	63.24 ± 36.96 ^c^	66.96 ± 2.25 ^c^
31	1540	13744-15-5	cubebene	citrus, fruity	RI/MS	5.52 ± 4.80 ^a^	ND ^b^	ND ^b^	ND ^b^
32	1553	469-61-4	(-)-α-cedrene	woody	RI/O/MS	ND ^a^	8.7 ± 0.96 ^b^	ND ^a^	ND ^a^
33	1585	13474-59-4	(E)-α-bergamotene	warm	RI/O/MS	54.61 ± 2.72 ^a^	44.9 ± 11.66 ^a^	11.81 ± 1.08 ^b^	23.42 ± 3.84 ^c^
34	1591	515-13-9	(-)-β-Elemene	sweet	RI/O/MS	56.50 ± 3.18 ^a^	50.9 ± 6.94 ^a^	15.99 ± 0.55 ^b^	13.05 ± 3.57 ^b^
35	1638		unknown		RI/MS	17.21 ± 5.56 ^a^	ND ^b^	ND ^b^	ND ^b^
36	1645	30021-74-0	γ-muurolene	herbal	RI/O/MS	8.30 ± 7.98 ^a^	ND ^b^	6.27 ± 4.45 ^a^	26.15 ± 0.87 ^c^
37	1657	18794-84-8	trans-β-Farnesene	woody, citrus, herbal, sweet	RI/MS	21.60 ± 2.55 ^a^	23.4 ± 7.00 ^a^	ND ^b^	ND ^b^
38	1664		unknown		RI/MS	ND ^a^	22.7 ± 9.95 ^b^	ND ^a^	ND ^a^
39	1718	495-60-3	zingiberene	spice fresh sharp	RI/O/MS	298.12 ± 37.39 ^a^	302.21 ± 64.91 ^a^	42.35 ± 8.71 ^b^	125.85 ± 28.22 ^c^
40	1726	495-61-4	unknown	balsamic woody	RI/MS	232.12 ± 20.91 ^a^	111.86 ± 96.97 ^b^	17.01 ± 2.20 ^c^	55.14 ± 3.50 ^d^
41	1739		unknown		RI/MS	ND ^a^	65.3 ± 20.46 ^b^	ND ^a^	ND ^a^
42	1771	644-30-4	unknown	herbal	RI/MS	564.20 ± 14.56 ^a^	565.82 ± 137.26 ^a^	104.65 ± 12.08 ^b^	251.63 ± 22.28 ^c^
43	1837	483-77-2	unknown	herb, spice	RI/MS	59.89 ± 2.5 ^a^	32.3 ± 9.08 ^b^	9.49 ± 2.93 ^a^	24.29 ± 9.29 ^b^
aromatic									
44	1029	108-88-3	Toluene	sweet	RI/O/MS	32.58 ± 8.15 ^a^	30.0 ± 3.47 ^a^	16.30 ± 3.34 ^b^	23.83 ± 1.23 ^c^
45	1207	95-63-6	1,2,4-Trimethylbenzene	plastic	RI/MS	4.37 ± 2.07 ^a^	ND ^b^	ND ^b^	ND ^b^
46	1125	108-38-3	m-Xylene	plastic	RI/O/MS	ND ^a^	8.4 ± 0.85 ^b^	6.37 ± 1.38 ^b^	ND ^a^
47	1166	95-47-6	o-Xylene	geranium	RI/O/MS	3 ± 0.33 ^a^	ND ^b^	ND ^b^	8.80 ± 4.19 ^c^
48	1256	99-87-6	p-Cymene	citrus	RI/O/MS	1212.91 ± 176.4 ^a^	908.03 ± 224.20 ^b^	725.57 ± 507.91 ^c^	731.00 ± 3.31 ^d^
49	1358	95-93-2	1,2,4,5-Tetramethylbenzene	rancid sweet	RI/MS	10.93 ± 3.16 ^a^	ND ^b^	ND ^b^	ND ^b^
50	1780	104-46-1	Anethole	sweet anise licorice medicinal	RI/MS	5.57 ± 1.93 ^a^	9.1 ± 2.38 ^b^	7.07 ± 2.75 ^a^	ND ^c^
51	1701	90-12-0	1-Methylnaphthalene	naphthyl chemical medicinal camphor	RI/MS	ND ^a^	15.5 ± 5.39 ^b^	ND ^a^	3.69 ± 0.89 ^a^
52	1846	91-57-6	2-Methylnaphthalene	sweet floral woody	RI/MS	15.19 ± 4.93 ^a^	10.2 ± 5.30 ^a^	140.80 ± 18.86 ^b^	ND ^c^
53	1870	94-59-7	Safrole	spicy	RI/O/MS	1183.47 ± 114.81 ^a^	1100.6 ± 363.06 ^a^	256.62 ± 40.63 ^b^	329.51 ± 2.18 ^b^
54	1944	120-58-1	Isosafrole (Controlled Chemical)	sweet sassafrass spicy	RI/MS	5.43 ± 2.67 ^a^	ND ^b^	ND ^b^	ND ^b^
55	2001	93-15-2	1,2-Dimethoxy-4-allylbenzene	sweet fresh warm spicy clove carnation cinnamon	RI/MS	629.17 ± 45.82 ^a^	661.22 ± 183.00 ^a^	ND ^b^	248.16 ± 9.78 ^c^
56	2146	607-91-0	Myristicin	spicy warm balsamic woody	RI/MS	927.43 ± 54.89 ^a^	973.65 ± 277.95 ^a^	185.01 ± 21.45 ^b^	328.50 ± 19.14 ^d^
57	2156	97-53-0	Eugenol	sweet, clove	RI/O/MS	51.19 ± 3.45 ^a^	ND ^b^	14.63 ± 2.79 ^c^	ND ^b^
58	2213	487-11-6	5-allyl-1,2,3-trimethoxybenzene	spice, flower	RI/MS	553.06 ± 16.46 ^a^	607.01 ± 193.07 ^a^	118.76 ± 17.70 ^b^	242.12 ± 22.34 ^c^
aldehyde									
59	964	110-62-3	Valeraldehyde	fermented bready fruity nutty berry	RI/MS	ND ^a^	ND ^a^	ND ^a^	0.44 ± 0.10 ^b^
60	1071	66-25-1	Hexanal	grass	RI/O/MS	22.83 ± 4.33 ^a^	41.9 ± 5.29 ^b^	27.57 ± 3.41 ^a^	35.30 ± 5.15 ^c^
61	1172	111-71-7	Heptanal	fresh, fatty, green, herbal	RI/MS	7.85 ± 1.58 ^a^	39.4 ± 54.44 ^b^	ND ^c^	ND ^c^
62	1277	124-13-0	Octanal	fatty	RI/O/MS	25.12 ± 3.62 ^a^	ND ^b^	ND ^b^	ND ^b^
63	1384	124-19-6	Nonanal	fresh	RI/O/MS	298.49 ± 35.41 ^a^	274.60 ± 55.28 ^a^	104.22 ± 24.45 ^b^	121.14 ± 11.17 ^b^
64	1517	100-52-7	Benzaldehyde	sharp, sweet, bitter, almond, cherry	RI/MS	47.37 ± 6.55 ^a^	42.8 ± 2.00 ^a^	51.21 ± 60.18 ^b^	25.41 ± 0.87 ^c^
65	1769	122-03-2	p-Isopropylbenzaldehyde	spicy, green, herbal	RI/MS	2.98 ± 0.37 ^a^	2.6 ± 0.82 ^a^	ND ^b^	ND ^b^
ester									
66	1345	103-09-3	2-Ethylhexyl acetate	earthy, herbal, undergrowth	RI/MS	37.44 ± 6.73 ^a^	ND ^b^	ND ^b^	ND ^b^
67	1572	76-49-3	Bornyl acetate	woody, pine, herbal, spice	RI/MS	154.48 ± 10.13 ^a^	129.59 ± 37.71 ^a^	51.54 ± 3.52 ^b^	63.45 ± 4.45 ^b^
68	1619	110-38-3	ethyl caprate	sweet, waxy, fruity, apple grape, oily, brandy	RI/MS	19.08 ± 3.32 ^a^	32.3 ± 15.78 ^b^	8.53 ± 0.46 ^c^	9.12 ± 2.28 ^c^
69	1629	150-84-5	Citronellyl acetate	green, rose, fruity, citrus, woody, fruit	RI/MS	73.87 ± 3.22 ^a^	72.0 ± 28.66 ^a^	25.68 ± 2.39 ^b^	35.58 ± 6.76 ^c^
70	1665	80-26-2	( + /-)-α-terpinyl acetate	herbal, lavender, citrus	RI/MS	310.29 ± 61.43 ^a^	ND ^b^	ND ^b^	ND ^b^
71	1711	141-12-8	Neryl acetate	rose, soapy, citrus, pear	RI/MS	64.97 ± 36.40 ^a^	60.4 ± 19.49 ^a^	10.03 ± 3.75 ^b^	20.19 ± 5.79 ^c^
ketone									
72	856	67-64-1	Acetone	solvent, ethereal, apple, pear	RI/MS	ND ^a^	ND ^a^	5.10 ± 1.89 ^b^	ND ^a^
73	1309	110-93-0	6-Methyl-5-hepten-2-one	citrus, musty,	RI/O/MS	36.76 ± 3.11 ^a^	27.9 ± 6.06 ^b^	44.99 ± 41.95 ^c^	16.56 ± 2.43 ^d^
74	1639	89-81-6	Piperiton	herbal, minty, camphor, medicinal	RI/MS	ND ^a^	99.7 ± 151.32 ^b^	ND ^c^	ND ^c^
terpene									
75	1048	79-92-5	Camphene	camphor	RI/MS	ND ^a^	ND ^a^	16.07 ± 13.69 ^b^	17.48 ± 0.12 ^b^
76	1088	127-91-3	β-pinene	camphor	RI/O/MS	179.37 ± 207.01 ^a^	1217.9 ± 169.21 ^b^	935.41 ± 166.65 ^c^	919.37 ± 57.82 ^c^
77	1124	13466-78-9	3-carene	pine	RI/O/MS	337.9 ± 72.66 ^a^	284.22 ± 42.93 ^b^	294.13 ± 32.84 ^b^	213.76 ± 34.51 ^b^
78	1158	99-86-5	p-mentha-1,3-diene	lemon	RI/O/MS	1418.14 ± 256.37 ^a^	1131.9 ± 371.75 ^b^	1111.79 ± 152.95 ^b^	760.87 ± 40.85 ^c^
79	1178	5989-27-5	( + )-Limonene	citrus, orange, fresh, sweet	RI/MS	212.14 ± 139.31 ^a^	ND ^b^	ND ^b^	ND ^b^
80	1186	138-86-3	DL-Limonene	citrus, herbal, terpene, camphor	RI/MS	158.36 ± 106.1 ^a^	1415.9 ± 1281.47 ^b^	1632.77 ± 233.11 ^b^	1644.62 ± 93.95 ^b^
81	1187	470-82-6	Cineole	herbal	RI/O/MS	127.86 ± 17.99 ^a^	540.02 ± 748.14 ^b^	123.76 ± 22.81 ^a^	65.99 ± 5.19 ^c^
82	1229	99-85-4	p-mentha-1,4-diene	oily, woody, terpene, lemo, herbal	RI/MS	2360.05 ± 356.15 ^a^	2033.6 ± 590.91 ^a^	1796.05 ± 251.18 ^b^	1161.38 ± 52.07 ^c^
83	1268	586-62-9	terpinolene	camphor	RI/O/MS	1084.81 ± 127.42 ^a^	209 ± 64.16 ^b^	433.93 ± 372.24 ^ab^	510.30 ± 29.11 ^c^
84	1459	17699-16-0	(1α,2α,5α)-2-methyl-5-(1-methylethyl)bicyclo [3.1.0]hexan-2-ol	woody, balsam	RI/MS	146.83 ± 20 ^a^	383.09 ± 32.25 ^b^	512.91 ± 583.52 ^c^	ND^d^
85	1495	3856-25-5	(-)-α-copaene	woody, spicy, honey	RI/MS	317.19 ± 29.09 ^a^	233.11 ± 68.25 ^a^	68.22 ± 48.39 ^b^	ND^c^
86	1604	87-44-5	Caryophyllene	spice	RI/O/MS	190.16 ± 20.13 ^a^	191.55 ± 56.34 ^a^	461.25 ± 545.27 ^b^	140.08 ± 2.22 ^c^
87	1648	118-65-0	(-)-isocaryophyllene	woody, spicy	RI/MS	4.31 ± 1.90 ^a^	ND ^b^	ND ^b^	ND ^b^
88	1677	6753-98-6	α-humulene	woody	RI/MS	59.70 ± 6.75 ^a^	50.2 ± 16.84 ^a^	ND ^b^	9.18 ± 6.36 ^c^
89	1761	483-76-1	unknown	thyme, woody, dry	RI/O/MS	34.28 ± 1.42 ^a^	33.9 ± 9.08 ^a^	8.73 ± 1.25 ^b^	21.17 ± 4.87 ^c^

^a^, ^b^, ^c^ and ^d^ mean the different significance of the data.

**Table 2:** Volatile compounds identified by SAFE/GC-O-MS during the storage period of cooked mutton balls.

No	RI	CAS No	Compounds	Perception	Identification	Relative Content (μg/g)			
						0 d	30 d	60 d	90 d
alcohol									
1	1462	3391-86-4	1-Octen-3-ol	mushroom	MS/RI	ND ^b^	ND ^b^	0.37 ± 0.16 ^a^	ND ^b^
2	1554	89-79-2	Isopulegol	minty, cooling	MS/RI	ND ^c^	0.24 ± 0.09 ^ab^	0.07 ± 0.02 ^a^	0.33 ± 0.13 ^b^
3	1559	78-70-6	Linalool	citrus floral, rose	MS/RI/O/	0.40 ± 0.02 ^a^	1.52 ± 0.19 ^b^	1.22 ± 0.31 ^a^	3.81 ± 0.28 ^c^
4	1629	562-74-3	Terpinen-4-ol	pepper, woody, earth, musty, sweet	MS/RI	3.13 ± 0.12 ^a^	2.53 ± 0.40 ^b^	8.93 ± 1.97 ^c^	15.11 ± 1.19 ^d^
5	1721	98-55-5	p-menth-1-en-8-ol	citrus	MS/RI/O/	0.33 ± 0.02 ^a^	1.61 ± 0.38	1.61 ± 0.38 ^b^	13.26 ± 0.39 ^c^
6	1729	507-70-0	((1S)-endo)-(-)-borneol	camphor	MS/RI/O/	0.10 ± 0.06 ^a^	3.54 ± 0.46 ^b^	0.55 ± 0.15 ^a^	3.23 ± 0.12 ^b^
7	1778	617-94-7	2-Phenyl-2-propanol	green, earthy	MS/RI/O/	0.70 ± 0.06 ^c^	7.13 ± 4.24 ^a^	1.48 ± 1.05 ^b^	11.32 ± 1.26 ^a^
8	1864	106-24-1	Geraniol	rose, waxy, citrus	MS/RI/O/	0.91 ± 0.32 ^a^	1.03 ± 0.84 ^a^	0.20 ± 0.04 ^a^	1.23 ± 0.06 ^a^
9	1869	1197-01-9	2-p-Tolylpropan-2-ol	sweet, fruity, cherry, camphor	MS/RI	ND ^c^	3.81 ± 1.24 ^a^	0.83 ± 0.17 ^b^	0.29 ± 0.29 ^a^
Sulfur compound									
10	1162	592-88-1	Diallyl sulfide	sulfurous, onion, garlic	MS/RI	1.24 ± 0.12 ^a^	0.08 ± 0.12 ^b^	ND ^c^	ND ^c^
11	1297	2179-58-0	Allyl methyl disulphide	garlic, onion	MS/RI/O/	2.95 ± 0.03 ^a^	3.09 ± 0.54 ^a^	1.17 ± 0.25 ^b^	0.25 ± 0.08 ^a^
12	1503	2179-57-9	Diallyl disulfide	onion, garlic	MS/RI/O/	9.67 ± 0.50 ^a^	7.09 ± 0.92 ^b^	7.22 ± 1.65 ^a^	1.60 ± 0.09 ^c^
olefin									
13	1226	555-10-2	p-mentha-1(7),2-diene	mint, terpentine	MS/RI	2.53 ± 0.18 ^a^	9.65 ± 1.18 ^b^	7.71 ± 1.76 ^c^	ND ^d^
14	1272	100-42-5	Styrene	sweet, balsam, floral, plastic	MS/RI	8.83 ± 0.56 ^a^	3.53 ± 0.72 ^b^	3.45 ± 0.74 ^c^	1.49 ± 0.19 ^d^
15	1458	1195-32-0	Alpha, para-Dimethylstyrene	phenolic	MS/RI/O/	ND ^c^	0.72 ± 0.26 ^a^	0.22 ± 0.05 ^b^	ND ^c^
16	1582	13474-59-4	(E)-α-bergamotene	woody, warm, tea	MS/RI	ND ^d^	0.18 ± 0.08 ^a^	0.70 ± 0.18 ^b^	2.22 ± 0.16 ^c^
17	1750	495-60-3	Zingiberene	spice, sharp	MS/RI/O/	0.08 ± 0.03 ^a^	3.38 ± 0.37 ^a^	1.19 ± 0.22 ^b^	17.02 ± 1.08 ^c^
18	1757	495-61-4	Unknown	balsamic, woody	MS/RI	ND ^d^	0.94 ± 0.14 ^b^	0.41 ± 0.08 ^a^	5.17 ± 0.60 ^c^

19	1054	108-88-3	Toluene	paint	MS/RI	44.31 ± 2.00 ^a^	15.57 ± 2.47 ^b^	39.11 ± 7.42 ^a^	1.01 ± 0.08 ^c^
20	1198	95-47-6	o-Xylene	geranium	MS/RI	1.91 ± 0.05 ^a^	ND ^c^	0.85 ± 0.19 ^b^	ND ^c^
21	1287	99-87-6	p-Cymene	fresh, citrus, terpene, woody, spice	MS/RI	0.83 ± 0.08 ^a^	2.15 ± 1.52 ^b^	2.44 ± 0.57 ^c^	ND ^d^
22	1802	644-30-4	1-methyl-4-(6-methylhept-5-en-2-yl)benzene	herbal	MS/RI	0.42 ± 0.02 ^a^	3.58 ± 0.41 ^b^	1.37 ± 0.26 ^a^	13.57 ± 9.15 ^b^
23	1855	104-46-1	Anethole	sweet, anise, licorice, medicinal	MS/RI	ND ^d^	0.54 ± 0.05 ^a^	0.08 ± 0.02 ^b^	0.70 ± 0.07 ^c^
24	1892	91-57-6	2-Methylnaphthalene	sweet, floral	MS/RI/O/	ND ^d^	0.71 ± 0.27 ^a^	0.12 ± 0.04 ^b^	1.20 ± 0.16 ^c^
25	1906	94-59-7	Safrole	sweet, warm, spicy, woody, floral	MS/RI	1.36 ± 0.16 ^a^	7.37 ± 0.65 ^b^	4.87 ± 0.97 ^c^	11.68 ± 1.49 ^d^
26	2046	581-42-0	2,6-Dimethylnaphthalene	grass	MS/RI	ND ^c^	0.71 ± 0.43 ^a^	ND ^c^	0.42 ± 0.10 ^b^
27	2250	487-11-6	5-allyl-1,2,3-trimethoxybenzene	spice, flower	MS/RI/O/	0.41 ± 0.03 ^a^	5.20 ± 0.43 ^a^	2.19 ± 0.39 ^a^	10.06 ± 1.36 ^b^
28	2298	607-91-0	myristicin	spicy, warm, balsamic, woody	MS/RI/O/	0.74 ± 0.05 ^a^	7.39 ± 0.61 ^b^	3.33 ± 0.56 ^a^	18.46 ± 2.39 ^c^
aldehyde									
29	1095	66-25-1	Hexanal	fresh, green, fatty, aldehydic, grass	MS/RI/O/	4.68 ± 0.20 ^a^	1.38 ± 0.23 ^b^	0.11 ± 0.02 ^c^	0.42 ± 0.12 ^c^
30	1411	124-19-6	Nonanal	fresh	MS/RI/O/	0.95 ± 0.05 ^a^	0.61 ± 0.08 ^b^	0.39 ± 0.09 ^c^	2.37 ± 0.12 ^b^
31	1548	100-52-7	Benzaldehyde	almond, cherry	MS/RI	0.32 ± 0.07 ^a^	0.26 ± 0.05 ^b^	0.25 ± 0.08 ^a^	ND ^c^
ester									
32	912	141-78-6	Ethyl acetate	fruity, sweet, green	MS/RI	0.25 ± 0.19 ^c^	0.52 ± 0.15 ^a^	0.36 ± 0.09 ^b^	ND ^d^
33	1044	2867-05--2	Alpha-thujene	woody, green, herb	MS/RI	1.75 ± 0.07 ^a^	1.28 ± 0.18 ^b^	0.92 ± 0.19 ^c^	ND ^d^
34	1610	76-49-3	Bornyl acetate	woody, pine, herbal, spice	MS/RI	0.13 ± 0.02 ^a^	0.79 ± 0.08 ^b^	0.42 ± 0.10 ^c^	4.07 ± 0.15 ^d^
35	1659	110-38-3	Ethyl caprate	sweet, waxy, fruity	MS/RI	ND ^d^	0.63 ± 0.17 ^a^	0.10 ± 0.04 ^b^	0.90 ± 0.03 ^c^
36	1725	80-26-2	(+/-)-α-terpinyl acetate	herbal, bergamot, lavender	MS/RI	0.30 ± 0.09 ^a^	0.74 ± 0.12 ^b^	0.41 ± 0.09 ^a^	4.91 ± 0.23 ^c^
ketone									
37	1679	98-86-2	Acetophenone	sweet, pungent	MS/RI/O/	2.69 ± 0.16 ^a^	5.49 ± 0.72 ^b^	5.48 ± 1.22 ^c^	8.43 ± 0.16 ^ab^
terpene									
38	1040	80-56-8	α-pinene	pine, earthy, woody	MS/RI/O/	0.39 ± 0.01 ^a^	17.61 ± 2.89 ^b^	10.81 ± 2.35 ^c^	ND ^a^
39	1082	79-92-5	Camphene	woody, camphor, terpenic	MS/RI	ND ^c^	0.68 ± 0.03 ^a^	0.11 ± 0.03 ^b^	ND ^c^
40	1124	127-91-3	β-pinene	pine, green	MS/RI/O/	10.22 ± 0.48 ^a^	17.46 ± 2.51 ^b^	14.47 ± 3.30 ^a^	ND ^c^
41	1166	13466-78-9	3-carene	pine, woody	MS/RI/O/	ND ^c^	2.52 ± 0.56 ^a^	2.03 ± 0.43 ^b^	ND ^c^
42	1196	99-86-5	p-mentha-1,3-diene	woody, lemon, herbal	MS/RI/O/	0.92 ± 0.08 ^a^	3.78 ± 0.82 ^b^	1.98 ± 0.43 ^a^	ND ^a^
43	1216	5989-27-5	(+)-Limonene	citrus, orange	MS/RI	3.56 ± 0.14 ^a^	11.73 ± 1.66 ^b^	9.62 ± 2.27 ^c^	ND ^d^
44	1264	99-85-4	p-mentha-1,4-diene	woody, terpene	MS/RI/O/	3.70 ± 0.28 ^a^	6.89 ± 0.95 ^b^	5.06 ± 1.19 ^a^	ND ^c^
45	1302	586-62-9	terpinolene	pine, citrus	MS/RI/O/	0.94 ± 0.09 ^a^	3.21 ± 0.61 ^b^	2.35 ± 0.53 ^c^	0.41 ± 0.19 ^a^
46	1527	3856-25-5	(-)-α-copaene	woody, spicy, honey	MS/RI	0.28 ± 0.00 ^a^	0.82 ± 0.17 ^b^	0.82 ± 0.17 ^c^	10.31 ± 0.32 ^d^
47	1638	87-44-5	Caryophyllene	sweet, woody, spice	MS/RI/O/	0.20 ± 0.01 ^a^	5.60 ± 0.19 ^b^	0.62 ± 0.14 ^c^	8.49 ± 0.24 ^d^
48	1710	6753-98-6	α-Humulene	woody	MS/RI	ND ^b^	0.70 ± 0.24 ^a^	0.08 ± 0.04 ^b^	1.10 ± 0.28 ^a^
49	1763	-	unknown	herbal, woody	MS/RI	ND ^c^	0.51 ± 0.13 ^a^	0.19 ± 0.04 ^b^	2.53 ± 0.57 ^a^
50	1935	128-37-0	2,6-Di-tert-butyl-4-methylphenol	camphor	MS/RI/O/	5.26 ± 0.31 ^a^	9.81 ± 13.80 ^a^	3.19 ± 0.60 ^a^	18.12 ± 2.56 ^b^
51	2025	108-95-2	Phenol	plastic, rubber	MS/RI	0.13 ± 0.00 ^a^	0.12 ± 0.02 ^b^	0.09 ± 0.03 ^a^	ND ^c^
52	2034	93-15-2	1,2-Dimethoxy-4-allylbenzene	sweet, fresh,	MS/RI	0.55 ± 0.03 ^a^	7.72 ± 10.64 ^a^	2.90 ± 0.56 ^a^	11.01 ± 1.35 ^b^
53	2195	97-53-0	Eugenol	sweet, spicy	MS/RI	0.17 ± 0.06 ^a^	0.79 ± 0.10 ^b^	0.42 ± 0.09 ^a^	5.54 ± 0.25 ^c^
54	2325	96-76-4	2,4-Ditert-butylphenol	phenolic	MS/RI	0.93 ± 0.06 ^b^	2.07 ± 0.10 ^c^	0.24 ± 0.09 ^a^	0.62 ± 0.34 ^ab^
other									
55	1440	104-90-5	5-Ethyl-2-methylpyridine	nutty, strong	MS/RI	0.03 ± 0.01 ^a^	ND ^b^	ND ^b^	ND ^b^
56	1484	17699-16-0	unknown	woody, balsam	MS/RI/O/	1.46 ± 0.11 ^a^	4.97 ± 0.7 ^b^	3.95 ± 0.96 ^c^	5.71 ± 0.38 ^c^
57	1647	107-92-6	n-Butyric acid	cheesy	MS/RI/O/	0.92 ± 0.35 ^a^	1.43 ± 0.58 ^b^	0.10 ± 0.04 ^b^	0.90 ± 0.03 ^c^
58	1679	98-86-2	Acetophenone	sweet, pungent	MS/RI/O/	2.69 ± 0.16 ^a^	5.49 ± 0.72 ^b^	5.48 ± 1.22 ^c^	8.43 ± 0.16 ^ab^

^a^, ^b^, ^c^ and ^d^ mean the different significance of the data.

**Table 3:** The result of DHDA of SPME/GC-O-MS.

NO.	RI	CAS No	Compound	Odor	FD Factor			
					0 d	30 d	60 d	90 d
A1	871	870-23-5	Allyl mercaptan	garlic	243	81	9	9
A2	1187	470-82-6	Cineole	mint	3	9	-	-
A3	1392	3391-86-4	1-Octen-3-ol	mushroom	9	9	9	-
A4	1495	78-70-6	Linalool	floral	27	3	3	1
A5	1834	106-24-1	Geraniol	waxy	27	-	-	-
A6	1262	2179-58-0	allyl methyl disulphide	garlic, onion	-	9	-	-
A7	1454	2179-57-9	Diallyl Disulfide	onion garlic	3	9	9	-
A8	1754	2050-87-5	Diallyl trisulfide	garlic onion	243	27	9	27
A9	1013	80-56-8	α-pinene	pine	27	9	1	1
A10	1088	127-91-3	β-pinene	resin	3	1	3	-
A11	1124	13466-78-9	3-carene	pine resin	3	3	3	3
A12	1188	555-10-2	p-mentha-1(7),2-diene	terpentine	9	-	-	-
A13	1214	3779-61-1	trans-Ocimene	sweet	-	1	3	1
A14	1268	586-62-9	terpinolene	pine	243	-	-	-
A15	1418	18368-95-1	p-menthatriene, p-mentha-1,3,8-triene	turpentine	9	-	-	-
A16	1428	1195-32-0	α,para-Dimethylstyrene	citrus	3	1	3	1
A17	1455	3856-25-5	(-)-α-copaene	spicy	27	-	-	9
A18	1591	515-13-9	(-)-β-Elemene	sweet	9	3	1	-
A19	1604	87-44-5	Caryophyllene	spice	3	-	-	-
A20	1761	483-76-1	unknown	medicine	9	9	1	9
A21	1256	99-87-6	p-Cymene	citrus	-	-	9	27
A22	1701	90-12-0	1-Methylnaphthalene	camphor	-	-	3	-
A23	1944	120-58-1	Isosafrole (Controlled Chemical)	sweet	3	3	9	3
A24	2001	93-15-2	1,2-Dimethoxy-4-allylbenzene	clove	-	9	-	-
A25	1619	110-38-3	ethyl caprate	grape	3	1	3	3
A26	1639	110-93-0	6-Methyl-5-hepten-2-one	mushroom	3	1	3	3

**Table 4:** The result of AEDA of SAFE/GC-O-MS.

No.	RI	CAS No	Compounds	Odor	FD Factor			
					0 d	30 d	60 d	90 d
B1	1040	80-56-8	α-pinene	pine	-	3	3	-
B2	1166	13466-78-9	3-carene	resin	-	3	1	-
B3	1196	99-86-5	p-mentha-1,3-diene	lemon	-	1	3	-
B4	1458	1195-32-0	α,para-Dimethylstyrene	pine	-	3	9	-
B5	1638	87-44-5	Caryophyllene	spice	3	27	9	1
B6	1750	495-60-3	zingiberene	sharp	1	3	1	-
B7	1095	66-25-1	Hexanal	fat	9	1	1	1
B8	1297	2179-58-0	allyl methyl disulphide	garlic	9	3	81	3
B9	1503	2179-57-9	Diallyl Disulfide	onion	27	81	9	3
B10	2298	607-91-0	myristicin	spicy	1	3	-	-
B11	1559	78-70-6	Linalool	floral	3	3	9	3
B12	1721	98-55-5	p-menth-1-en-8-ol	lilac	1	1	1	3
B13	1729	507-70-0	((1S)-endo)-(-)-borneol	camphor	-	3	-	-
B14	1778	617-94-7	2-Phenyl-2-propanol	green	-	3	1	1
B15	1864	106-24-1	Geraniol	sweet	9	9	9	3
B16	1484	17699-16-0	unknown	balsam	3	3	9	3
B17	1647	107-92-6	n-Butyric acid	sharp	3	3	3	3
B18	1679	98-86-2	Acetophenone	almond	-	3	1	-
B19	1892	91-57-6	2-Methylnaphthalene	floral	-	-	9	1
B20	2250	487-11-6	5-allyl-1,2,3-trimethoxybenzene	spice	-	3	-	9

**Table 5:** OAVS OF odor-active compounds in cooked mutton meatballs.

No.	CAS No	Compounds	Odor	OAV				Threshold Value (μg/kg)
				0 d	30 d	60 d	90 d	
1	470-82-6	Cineole	Herbal, medicinal	98.35	415.4	95.2	50.76	1.3
2	3391-86-4	1-Octen-3-ol	mushroom	25.99	26	29.78	18.6	2
3	78-70-6	Linalool	floral	56.46	50.67	19.82	27.28	6
4	10152-76-8	Allyl methyl sulfide	Garlic, onion	244.1	134.8	77.14	76.24	0.5
5	2179-57-9	Diallyl Disulfide	garlic	858.24	1380.08	822.58	633.94	1.3
6	80-56-8	α-pinene	Pine, woody	16.6	10.4	7.65	8.42	100
7	127-91-3	β-pinene	Woody, pine	1.28	8.7	6.68	6.57	140
8	3856-25-5	(-)-α-copaene	woody spicy honey	6.53	-	-	24.63	6
9	93-15-2	1,2-Dimethoxy-4-allylbenzene	sweet	9.25	9.72	0	3.65	68
10	66-25-1	Hexanal	grassy	104	30.67	2.44	9.33	45
11	124-19-6	Nonanal	Waxy, aldehydic	27.14	17.43	11.14	67.71	35
12	607-91-0	myristicin	spicy	24.67	246.33	111	615.33	30
13	507-70-0	((1S)-endo)-(-)-borneol	Pine, woody	1.92	68.08	10.58	62.12	52
14	128-37-0	2,6-Di-tert-butyl-4-methylphenol	mild phenolic camphor	5.26	9.81	3.19	18.12	100
15	107-92-6	n-Butyric acid	sweaty	920	1430	100	900	1
16	98-86-2	Acetophenone	pungent	15.82	32.29	32.24	49.59	170
17	91-57-6	2-Methylnaphthalene	sweet	-	177.5	30	300	4

## Data Availability

All data included in this study are available upon request by contact with the corresponding author.
